# Estrogenic, cytotoxic, proliferative effects of mycotoxins in human breast cancer cell line models: a systematic review

**DOI:** 10.3389/fpubh.2026.1950615

**Published:** 2026-10-30

**Authors:** Sivakumar Sasidharan, Piplani Naina, Dubey Jyoti, Taruna Arora, Archana Burman, Savita Bansal, Sivakumar Gowardhan, Abha Mishra, Nilesh Chandra

**Affiliations:** 1 Division of Discovery Research, Indian Council of Medical Research, New Delhi, India; 2 Department of Biochemistry, Institute of Home Economics, University of Delhi, New Delhi, India; 3 Department of Prosthodontics and Crown & Bridge, Ragas Dental College and Hospital, Tamil Nadu Dr. M.G.R Medical University, Chennai, Tamil Nadu, India; 4 Research and Development, Diabetes Research Foundation, New Delhi, India

**Keywords:** breast cancer, cytotoxicity, estrogen receptor, estrogenic activity, mycotoxins, systematic review, zearalenone

## Abstract

**Objective:**

To systematically review the estrogenic, proliferative, and cytotoxic effects of mycotoxins across human breast cell line models.

**Methods:**

A systematic review was conducted across five primary databases, MEDLINE (via PubMed), Scopus, EMBASE, Web of Science, and Google Scholar, covering literature published up to December 2025 in adherence to PRISMA 2020 guidelines. Screenings were performed independently by two reviewers, and data regarding experimental models, exposure concentrations, durations, and phenotypic or mechanistic outcomes were systematically extracted.

**Results:**

Forty-one studies spanning 20 mycotoxin classes across 11 cell lines revealed a clear receptor-dependent dichotomy. ER+ Models: Zearalenone (ZEN) and alpha-zearalenol acted as potent estrogen agonists. Alpha-zearalenol showed the highest relative proliferative potency (RPP = 7, normalized relative to 17β-estradiol assigned a reference baseline value of 1.0). TNBC/ER- Models: Lines were resistant to hormone-driven growth but highly vulnerable to cytotoxins. Aflatoxin B1 collapsed cell viability to 31.1% at 9.6 μg/mL. Gliotoxin (IC50: 38–985 nM) and Verrucarin A (IC50: 0.29–0.41 μM) displayed severe toxicity.

**Conclusion:**

Mycotoxins exert diverse, receptor-dependent biological impacts rather than a uniform pharmacological effect. Dietary endocrine disruptors like ZEN act as potent environmental xenoestrogens *in vitro*, highlighting a strong mechanistic plausibility for tumor promotion in hormone-sensitive breast tissues. Future research must prioritize 3D organoids, co-exposure mixtures, and epidemiological tracking. However, given the predominantly medium risk of bias across the evaluated *in vitro* literature, these findings should be interpreted with methodological caution.

**Systematic review registration:**

https://www.crd.york.ac.uk/PROSPERO/view/CRD420261346524; https://osf.io/qh8w6/files/7kce6.

## 1 Introduction

Breast cancer is the most frequently diagnosed cancer among women worldwide and is the leading cause of cancer-related mortality (1–3). According to the estimates from the GLOBOCAN 2022 data, compiled by the International Agency for Research on Cancer, approximately 2.3 million new breast cancer cases were reported in 2022, accounting for about 11.6% of all cancer diagnoses and representing the highest contributor to the female cancer burden in 157 of 185 countries (4). According to the World Health Organization (2024), breast cancer was responsible for 670,000 deaths globally, 6.9% of all cancer-related deaths, making it the fourth leading cause of cancer death overall and the leading cause of cancer death in women in the same year. With 985,817 new cases, Asia has the highest absolute incidence burden in the region, followed by Europe (557,532) and North America (306,307). Africa has a disproportionately high mortality-to-incidence ratio (MIR) of 0.510, which reflects serious deficiencies in early detection and treatment infrastructure (5).

Significantly, estimates show that by 2050, more than 6 million breast cancer cases will be recorded worldwide, with Asia experiencing the largest absolute increase at 2 million new cases (5). The urgent need to discover and define all modifiable environmental and nutritional risk factors that may contribute to the development, progression, and therapeutic resistance of breast cancer is highlighted by this increasing worldwide burden. Approximately 1 in 12 women in nations with a very high Human Development Index (HDI) are diagnosed with breast cancer during their lifetime, but only 1 in 71 die from it. According to WHO, 2024 this lower mortality rate reflects better access and early treatment. Conversely, only 1 in 27 women in low-HDI nations receive a diagnosis, which is likely due to underdiagnosis rather than a really lower incidence (4). These disparities necessitate immediate investigation of region-specific risk factors, such as dietary exposure to environmental contaminants that are common in low and middle-income regions, where mycotoxin contamination of staple food crops is endemic.

Breast cancer is a diverse group of molecular subtypes that are primarily identified by the expression status of Human epidermal growth factor receptor 2, progesterone receptor (PR), and estrogen receptor alpha (ERα). This is the most common subtype, which accounts for about 70–75% of all breast cancer diagnoses worldwide (6). Because this subtype is estrogen-dependent, estrogen receptor activation drives transcriptional programs that control cell cycle progression, anti-apoptotic signaling, and metastatic potential, rendering ER-positive (ER+) breast cancer susceptible to exogenous estrogenic stimuli. This receptor-driven sensitivity is of central relevance to the study of mycotoxins, specifically, mycotoxins that structurally mimic 17β-estradiol (E2) can bind to ERs and initiate transcriptional cascades (7, 8). Because primary breast cancer risk factors are heavily tied to estrogen expression, reproductive history, and genetics (3, 9–13), this receptor-driven sensitivity makes breast tissue highly vulnerable to mycotoxins, which trigger various biological effects that are likely linked to cancer development (2).

Mycotoxins are toxic secondary metabolites synthesized by filamentous fungi under specific environmental conditions by several mold genera. The primary mycotoxin-producing genera include Penicillium, Fusarium, and Aspergillus species, which contaminate numerous agricultural products worldwide (14, 15). Warm and humid conditions provide optimal environments for mold proliferation across staple commodities, such as cereal grains, legumes like groundnuts, tree nuts, and different varieties of dried fruits and spices. The Food and Agriculture Organization (FAO) has estimated that approximately 25% of the world's food crops are contaminated with mycotoxins at detectable levels, a figure that substantially underestimates the global population's total dietary burden when agriculturally prevalent low-level contamination is considered (16). Principal mycotoxins classes include Zearalenone (ZEA) (from Fusarium spp.), aflatoxins (AFB) (from Aspergillus spp.), ochratoxin A (OTA) (from Aspergillus and Penicillium spp.) and fumonisins (from Fusarium spp) (15). The primary route of human mycotoxin exposure is the consumption of contaminated foods that have been infected by mold. Many developing regions majorly rely on diets that include cereals and nuts, which are a common dietary exposure (7, 17, 18).

One notable example is Aflatoxin B1 (AFB1), which becomes bioactivated through the action of liver-based cytochrome P450 enzymes. A critical step in the progression of malignancy is the ability of AFB1 to specifically target and mutate the TP53 tumor suppressor gene, thereby disrupting essential cellular regulation and promoting oncogenesis (17). Many mycotoxins have mutagenic and genotoxic properties, with oxidative stress being a common mechanism. During metabolism, several mycotoxins generate reactive oxygen species (ROS), which create an imbalance that damages DNA, lipids and proteins (17), leading to dysregulation and cancer development. Prolonged oxidative stress may activate inflammatory pathways and promote genetic mutations, contributing to the onset and development of cancer (2). Beyond their genotoxic and oxidative effects, mycotoxins can compromise two of the cell's most fundamental regulatory processes, i.e., apoptosis and its response to hormonal signals. For example, Fumonisim B1 interferes with cells' management of sphingolipid signaling, effectively blocking the apoptotic machinery that eliminates damaged cells, allowing them to persist and accumulate (19). Hormonal pathways are also impacted by mycotoxins like zearalenone (ZEA), which structurally resembles estrogen closely enough to bind estrogen receptors in various tissues and trigger the same biological responses as the body's own hormones. Endocrine-disrupting chemicals (EDCs) capable of binding with estrogen receptors in breast tissues have been shown to activate oncogenic transcriptional programmes promoting tumor proliferation (2, 20). Chronic exposure could potentially function as a prolonged estrogenic stimulus due to the prevalence of ZEA-contaminated foods (grains, dairy, etc.) and ZEA resistance and degradation (21).

Despite the established biological plausibility of *in vitro* evidence examining mycotoxin-breast cancer cell interactions, the existing literature remains fragmented across diverse experimental paradigms, cell line models, concentration ranges, and mechanistic endpoints. This systematic review was therefore undertaken to comprehensively identify and synthesize all available *in vitro* evidence examining the estrogenic, proliferative, and cytotoxic effects of mycotoxins on human breast cancer cell lines. This review aims to characterize the range of mycotoxins studied and document the phenotypic biological outcomes, including cell viability, proliferation, apoptosis, cell cycle regulation and receptor modulation, induced by each mycotoxin class across ER-positive (ER+), ER-negative (ER-), TNBC, and normal breast cancer epithelial cell models, to identify and compare the mechanistic signaling pathways.

## 2 Methodology

### 2.1 Study design, review questions and PO framework

This investigation was conducted as a systematic review, following “Preferred Reporting Items for Systematic Reviews and Meta-Analyses (PRISMA) 2020 guidelines” (22–24) (Supplementary Table S1). This study summarizes data from the primary cell line studies investigating the effect of mycotoxin on breast cancer. A PO framework was used to formulate the review questions.

Q1: Which major mycotoxin classes (e.g. aflatoxins, fumonisins, ochratoxins, zearalenone) have been studied in relation to breast cancer cell line models?
Q2: What are the estrogenic, cytotoxic, and proliferative effects of mycotoxins that influence breast cancer?

PO Framework: Studies evaluating mycotoxin effects on breast cancer cell line models ERP, ERN, and TN were included. The study outcome is focused on estrogenic, cytotoxic, and proliferative effects of any type of mycotoxin exposure, namely, aflatoxins, ochratoxin A, fumonisin, deoxynivalenol, and zearalenone, etc. To ensure transparency and methodological rigor, the review protocol was registered with the Open Science Framework (25) and the international systematic review registry PROSPERO (CRD420261346524) before data commencement.

### 2.2 Search strategy

A comprehensive search strategy was developed and systematically applied across all primary databases to identify relevant evidence on the biological effects of mycotoxins in human breast cancer cell line models, and to establish methodological standards for conducting systematic reviews (22). Searches were executed across five primary databases- MEDLINE (via PubMed), Scopus, EMBASE, Web of Science, and Google Scholar, without restrictions on the publication year. Google Scholar was included as a supplementary primary database specifically to capture relevant gray literature, early-access institutional repositories, and non-indexed regional publications pertinent to endemic mycotoxin exposure. Language limits were applied where required by database-specific filters. Controlled vocabulary terms (MESH headings) were combined with relevant free-text keywords related to Mycotoxins and Breast cancer using Boolean operators (“AND,” “OR”) to maximize sensitivity while ensuring precision. For mycotoxins, broad terms were “mycotoxins,” “fungal toxin”) were combined with some specific toxins and classes (for example, “aflatoxin,” “ochratoxin”). For Breast cancer, filters and text terms were used, such as “breast cancer”, TS (“breast malignant neoplasm”) and “breast malignancy.” For the research design, majorly breast cancer cell lines were prioritized using specific breast cancer cell line filters, such as “breast cell line,” “malignant cell line,” “cell line” OR “cell lines” OR “*in vitro*” OR “MCF-7” OR “T47D” OR “MDA-MB-231” in google scholar, TITLE-ABS-KEY (“cell lines”) in SCOPUS and cell line [MESH] in MEDLINE are used. To maintain transparency and capture any eligible studies not indexed electronically, reference lists of all retrieved articles were hand-searched. Full details of all database-specific search strategies were presented in Supplementary Table S2.

### 2.3 Eligibility criteria

Studies were considered appropriate for inclusion if they reported experimental *in vitro* investigations examining the biological effects of one or more mycotoxins in human breast cancer or mammary epithelial cell lines, and if they were published in the English language. Both hypothesis-driven experimental studies and observational *in vitro* investigations were eligible, provided they include quantitative outcome data relevant to breast cancer biology (22, 23).

Studies were omitted if they were review articles, conference abstracts, letters, editorials, or commentaries or if they did not involve breast tissue, breast cancer, or mammary cell models. These eligibility criteria were pre-specified and consistently applied throughout the screening process.

### 2.4 Study selection

The study selection procedure adhered to PRISMA principles to ensure a transparent and reproducible workflow (22, 23). All the records retrieved from the different search sources were imported into a reference management software (i.e., CADIMA), where automated and manual procedures were applied to identify and remove duplicate entries. After the duplicates removal, the remaining records underwent title and abstract screening, which was performed by two reviewers (NP & JD) independently using the pre-defined eligibility criteria. Studies were advanced to full-text screening when the available information confirmed or was sufficient for the eligibility criteria. Full-text articles were further independently assessed by both reviewers (NP & JD). During this phase, 32 studies were excluded primarily due to missing comparators (*n* = 20) or the failure to report valid quantitative outcome metrics (*n* = 12). Discrepancies were settled through mutual agreement with the involvement of a third reviewer (SS) when required. The complete study selection process was documented using a PRISMA flow diagram (22, 23) (Figure 1).

**Figure 1:**
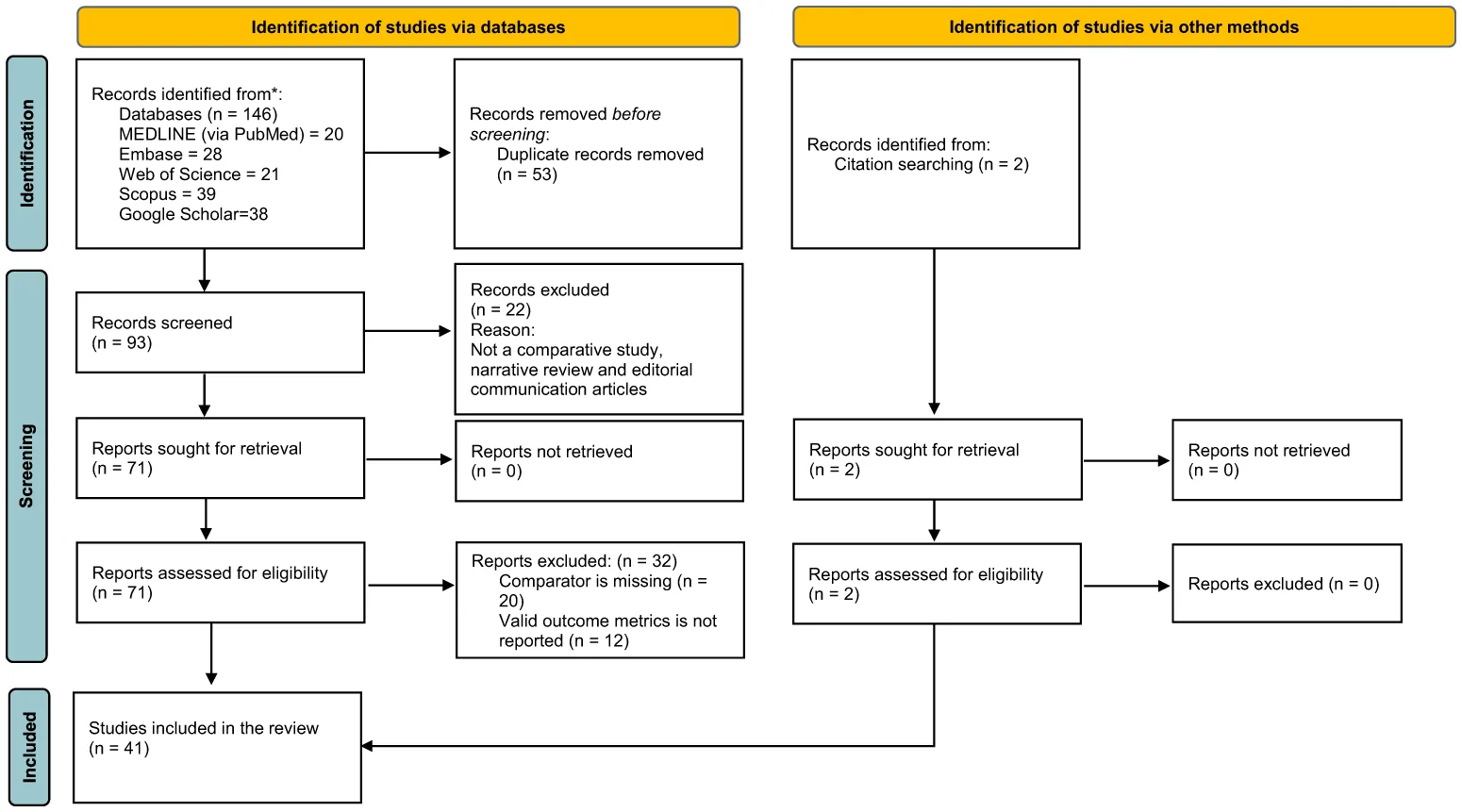
PRISMA flow chart of study selection.

### 2.5 Data extraction

Data from each eligible observational and experimental study, after title and abstract, and full text screening, were extracted using the predefined data extraction columns to ensure consistency and to preserve the original evidence base, in accordance with the recommended data extraction practices (24). For every review, information was captured on the setting and characteristics of the breast cancer experimental model, including breast cancer cell line (e.g., ERP, ERN, and TN breast cancer cell line models), with the name of the specific cell line. Information related to the mycotoxin exposure was comprehensively recorded, including the type of mycotoxin, culture medium used, and the control culture mediums. Exposure parameters documented, including the exposure level or the concentration, and the frequency of administration.

In order to determine the biological and carcinogenic significance of mycotoxin exposure, outcome-related variables were assessed. These variables included any reported quantitative outcome value, as well as the outcome related to breast cancer that was evaluated, such as cell proliferation, apoptosis, oxidative stress, DNA damage, or hormone receptor modulation. The overall effect of the mycotoxin on processes linked to breast cancer was also assessed in each study, and any mechanistic pathways found, such as estrogenic activity, cytotoxic activity, and proliferative activity, were recorded. To evaluate potential conflicts of interest, information about study funding sources was also extracted when it was available. Both reviewers extracted data independently for each included study, and disagreements were settled through discussion to ensure accuracy and comprehensiveness as recommended by PRISMA guidance (22, 23).

Due to the substantial methodological heterogeneity across the included literature, encompassing variations in exposure concentrations (ranging from picomolar to micromolar levels), exposure durations, and differing cell line phenotypes, quantitative meta-analysis was precluded. Consequently, a structured narrative synthesis was conducted. Evidence was systematically stratified first by the hormone receptor status of the experimental models (ER-positive, TNBC/ER-negative, and non-tumorigenic), and subsequently by the specific mycotoxin class to accurately isolate receptor-dependent phenotypic responses.

### 2.6 Risk of bias assessment

To evaluate the methodological quality of the included studies, risk of bias (RoB) was independently assessed by two reviewers. Due to the non-availability of universally accepted appraisal tools specifically built for general cellular models (26), the Quality assessment tool for *in vitro* Studies (QUIN tool) was adapted for evaluation (27). Studies were evaluated across key domain criteria, including clarity of research objectives, detailed description of experimental protocols/cell culture parameters, inclusion of appropriate negative/positive controls, outcome measure standardization, statistical analysis transparency, and clear presentation of results. Each domain was scored to categorize studies as having a low, medium, or high overall risk of bias, with discrepancies between reviewers resolved by consensus.

## 3 Result

### 3.1 Search results

A total of 148 papers were identified across all the databases. After duplicate removal, 93 unique papers underwent title and abstract level screening by two independent reviewers (NP and JD) using CADIMA. Additionally, 2 records were identified via citation searching, bringing the total to 95 unique papers evaluated. This screening resulted in the exclusion of 22 papers. The remaining 73 papers were analyzed at full text screening, out of which 32 were excluded that did not meet the eligibility criteria. In total, 41 studies were included in qualitative data synthesis. Any disagreement at either stage was resolved through consultation with the third reviewer (SS). The level of agreement calculated through Cohen's kappa between the two authors (NP and JD) was 0.87 for titles and abstracts and 0.94 for full texts.

### 3.2 Characteristics of included studies

The review incorporated 41 studies, providing a thorough summary of the effects of mycotoxins on human breast cancer and normal epithelial cell line models. Most studies used estrogen-receptor-positive (ERP) luminal A cell lines to accurately replicate the hormone-dependent characteristics of breast cancer, with MCF-7 (3, 14, 28–48) being the most commonly used model. There were also other luminal A lines represented, including T47D and ZR-75-1 (45, 49–52). The included studies also frequently used triple-negative (TN) breast cancer cell lines, such as MDA-MB-231, MDA-MB-468, BT-20, and BT-549 (36, 38, 45, 49, 50, 52–58), as cell line models to study biological pathways that function independently of estrogen receptor signaling. Additionally, several studies used non-tumorigenic epithelial cell lines such as MCF-10A, 184A (49, 59, 60) to provide comparative baselines for normal responses and specific tumor susceptibility, summarized in Supplementary Tables S3, S4.

A wide spectrum of mycotoxins was tested. Zearalenone and its biotransformed metabolites, including alpha and beta- zearalenol, zearalanone, glucoside conjugates, and hydrolysed metabolites, were the most extensively studied, reflecting their strong estrogenic potential in ERP cell lineages (3, 28, 29, 31, 32, 35, 36, 39, 42, 46, 50–52, 56, 61, 62). Other notable toxins included aflatoxin B1 (3, 30, 59), ochratoxin A (40, 54, 58), fusarin C (41), gliotoxin (45), verrucarin A (37, 53), brefeldin A (63), patulin (43), fusaric acid (48), alternariol (33, 60), sterigmatocystin (58), beauvericin, and enniatins (54), each interrogated for distinct cytotoxic, apoptotic, or proliferative effects as summarized in Table 1 and Figure 2.

**Table 1:** Biological outcomes and mechanistic pathways of mycotoxins in breast cancer cell lines.

Mycotoxins	Primary biological outcomes	Quantitative highlights (efficacy & potency)	Identified mechanistic pathways	Supporting citations
Zearalenone (ZEN) & Metabolites	Estrogenic proliferation, anti-apoptotic activity, and biphasic tumor promotion.	• Viability increased by +29.2% at 2 nM and +190.4% at 96 nM.	ERα/ERβ agonism; transcription of estrogen-responsive genes; Ras/ERK cascade activation; Bcl-2 upregulation.	(3, 28, 29, 31, 32, 34–36, 38, 39, 42, 46, 47, 50–52, 54, 60, 62, 64, 71)
		• Accelerated G1 to S phase transition (S-phase increased from 20% to 40%).		
α-Zearalenol (α-ZOL)	Exceeds parent ZEN in estrogenicity; strongest proliferative potency among derivatives.	• Proliferative Effect (PE) = 2.6.	High-affinity binding to ERα, mimicking 17β-estradiol (E2); stereospecific reduction of C-6 keto group.	(3, 30, 34, 36, 38–40, 42, 45, 50, 56, 64)
		• Relative Proliferative Potency (RPP) = 7.		
Hydrolyzed ZEN (HZEN & DHZEN)	Negligible estrogenic and proliferative activity.	Estrogenic potency is ~50 to 10,000 times less than intact ZEN; fails to induce proliferation.	Loss of the intact lactone ring abolishes ERα binding capacity.	(28, 56)
Zearalenone-14-glucoside	Masked mycotoxin; converted into active metabolites by cancer cells.	Causes no direct toxicity up to 1 mM, but is enzymatically converted to active ZEN, α-ZOL, and β-ZOL over 2–6 hours.	Enzymatic hydrolysis by MCF-7 cells unmasks estrogenic potential, activating ER signaling.	(61, 63)
Aflatoxin B1 (AFB1)	Dose-dependent cytotoxicity, genotoxicity, and apoptosis.	• Reduced MCF-7 viability to 31.1%at 9.6 μg/mL.	CYP450-mediated bioactivation forming DNA adducts; TP53 mutation; intracellular ROS accumulation.	(3, 30, 32, 33, 48, 59)
		• Induced apoptosis rates up to 46.4% at highest doses.		
Gliotoxin	Strong broad-spectrum antiproliferative effect.	IC50 values ranging from 38 to 985 nM across six distinct breast cancer cell lines.	Dual inhibition of farnesyltransferase (FTase) and geranylgeranyltransferase I (GGTase I).	(45)
Verrucarin A	Pro-apoptotic and cytotoxic activity.	• IC50 concentrations of 0.41 and 0.29 μM.	Loss of mitochondrial membrane potential (Δψm); ROS generation; p38 MAPK activation; increased Bax/Bcl-2 ratio.	(37, 53)
		• Synergistically enhances TNF-α-induced apoptotic responses.		
Brefeldin A	Preferential induction of anoikis and reduced cancer stem cell potential.	Exceptionally high potency with EC50 ≈ 0.016 μg/mL in TNBC (MDA-MB-231) suspension cultures.	ER-stress activation (increased IRE1α, PERK, CHOP); PARP cleavage; suppression of MMP-9.	(63)
Ochratoxin A (OTA)	Cytotoxicity, oxidative stress, and structural-dependent resistance.	• Monolayer IC50 ≈ 3 μM at 24 h.	ROS-mediated oxidative stress; intrinsic mitochondrial apoptosis; pH-dependent transport via mBcrp/hBCRP.	(40, 48, 49, 54, 58, 64)
		• Transporter-dependent growth inhibition IC50 ≈ 12.8–16.4 μM.		
Alternariol (AOH) & Altertoxins (AME, ATX-I, ATX-II)	Indirect estrogen-like proliferative effects (low doses) and genotoxicity (high doses).	• Induced CYP1 enzyme activity to ~190% of controls.	Aryl Hydrocarbon Receptor (AhR) activation; shifts estrogen metabolism toward genotoxic catecholestrogens.	(28, 33, 58, 60)
		• Altertoxin I + II exhibited synergistic activation (CI50 = 0.66).		
Wortmannin	Tumor growth inhibition and pro-apoptotic induction.	• Apoptosis increased from 1.75% (1 nM) to 25.3% (50 nM).	Targeted inhibition of PI3K/Akt and NF-κB signaling axes; suppression of Epithelial-Mesenchymal Transition; microRNA expression dysregulation.	(47, 56)
		Downregulation of oncogenic miR-21 and Vimentin protein expression; upregulation of NME1, miR-19a/b, miR-22.		
Patulin	Time-dependent, selective cytotoxicity.	• IC50 ≈ 25 μM in MCF-7 cells.	Intracellular ROS detection; time-dependent apoptotic cell death.	(43, 48, 52, 58)
		• At 100 μM, cancer viability fell to 12%, while normal cell viability remained at 36%.		
(5Z)-7-Oxozeaenol	ROS accumulation and cell cycle arrest in TNBC models.	Induced G1-phase cell cycle arrest in ~64% of treated MDA-MB-231 cells.	TAK1 inhibition; suppression of NF-κB proteins (p65, p50) independently of ER agonism.	(41, 65)
Beauvericin (BEA) & Enniatin B (ENN B)	Strong dose-dependent cytotoxicity.	24 h IC50 values: ENN B (0.15 μM) > BEA (1.8 μM).	Ionophoric disruption of cellular homeostasis.	(54, 64)
Fusaric Acid	Time-dependent inhibition of proliferation and cell cycle arrest.	Induced G2/M phase arrest at 500 μM; reduced colony formation.	Endoplasmic Reticulum (ER) stress pathway triggering.	(48)
Cytochalasin B	Selective cytotoxicity and mitochondrial dysfunction.	• IC50 ≈ 12.5 μMin ZR-75-1 cells (98.2% cell death).	Intracellular Ca2+ elevation and loss of mitochondrial membrane potential (Δψm).	(49)
		• No toxicity observed in normal MCF-10A cells.		
Phomopsin	Cytotoxicity via suppression of cellular synthesis.	Reduced thymidine incorporation to 41% and glycine incorporation to 36% at 10-6 M.	Severe inhibition of DNA replication and total protein synthesis.	(44, 62)
Fusarin C	Moderate estrogenic proliferation and high-dose toxicity.	Estrogenic activity with EC50 = 890 nM; high-dose toxicity at IC50 ≈ 46.8 μM.	Estrogenic activity confirmed via CALUX reporter assay.	(39)
α- and β-Amanitin	High potency cellular destruction via transcription block.	α-Amanitin (LD50 = 1 μg/mL) showed significantly higher potency than β-Amanitin (LD50 = 10 μg/mL).	Direct inhibition of RNA polymerase II, halting total protein synthesis.	(34, 53)
10,11-Dehydrocurvularin, Galiellalactone & Curvularins	Antiproliferative, anti-metastatic, and pro-apoptotic effects in TNBC.	Dose-dependent reduction in viability (2–8 μM) and severe inhibition of migration/invasion.	Suppression of TGF-β-induced vasculogenic mimicry; PARP/caspase-3 cleavage.	(35, 59, 69)
Sterigmatocystin (STE)	Time and architecture-dependent cytotoxicity.	Exhibits time-dependent decreases in IC50 (24 to 72h); 3D spheroids are markedly more resistant than 2D cultures.	Disruption of ATP synthesis and cellular metabolism.	(48, 58)

**Figure 2:**
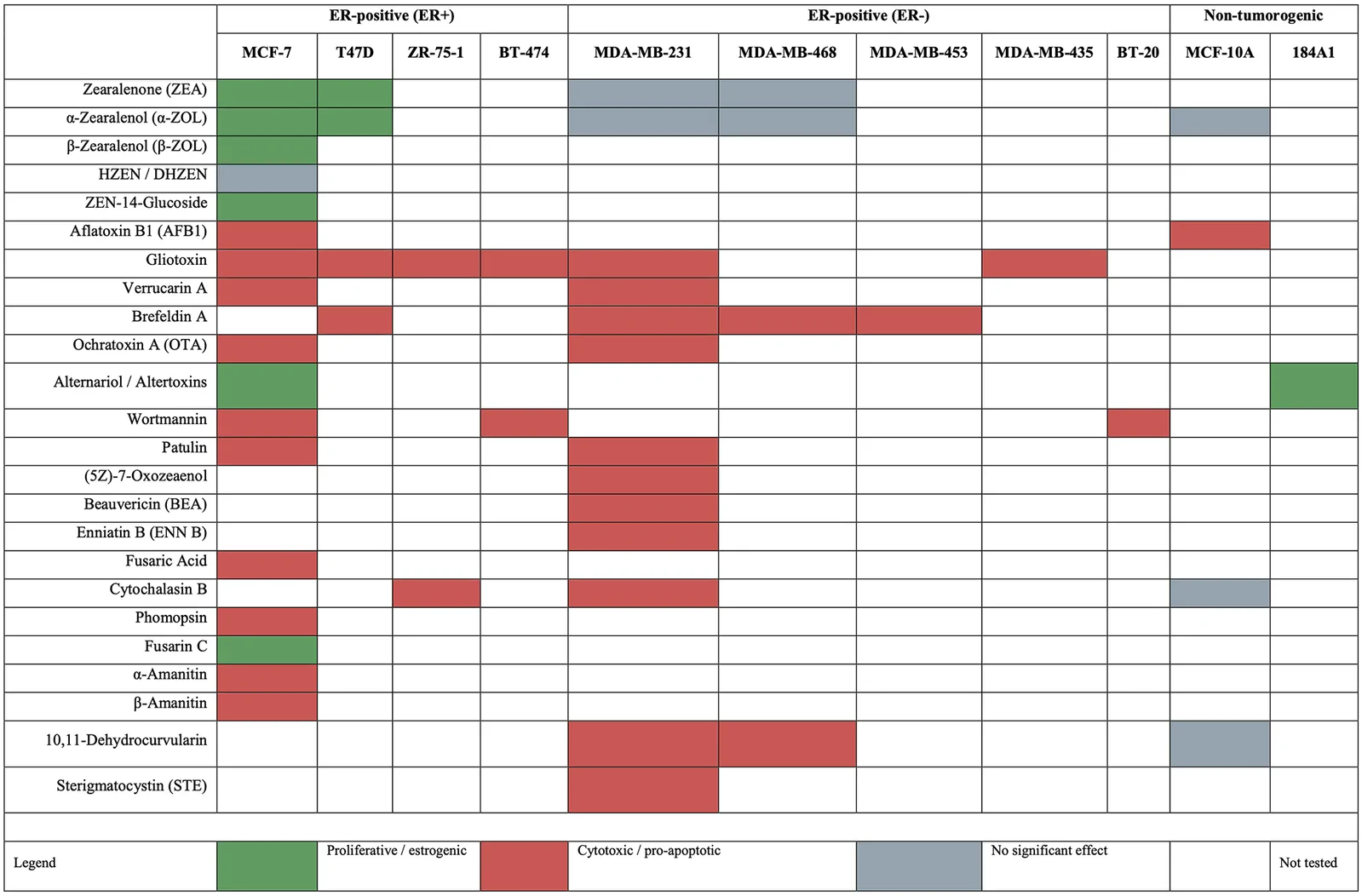
Heatmap of mycotoxin biological effects across human breast cancer cell line models. The biological effects of 24 mycotoxin classes across 11 human breast epithelial cell line models, compiled from 41 studies (1978–2025). Cell lines are grouped into three receptor-defined categories: (ER-positive (MCF-7, T47D, ZR-75-1, BT-474), TNBC/ER-negative (MDA-MB-231, MDA-MB-468, MDA-MB-435, BT-20), and non-tumorigenic (MCF-10A, 184A1). Each cell is color-coded to reflect the predominant observed response: green (proliferative/estrogenic), red (cytotoxic/pro-apoptotic), gray (no significant effect), or blank (not tested). The heatmap reveals a clear receptor-dependent pattern. ZEA mycotoxins elicit proliferative responses exclusively in ER+ lines, consistent with ER-alpha agonism, while showing no activity in ER-negative or non-tumorigenic models. Most estrogenic mycotoxins display cytotoxic activity predominantly in TNBC lines, with several exhibiting tumor-selective effects relative to non-tumorigenic controls. Fusarin C is notable for a biphasic dose-response (estrogenic at low concentrations, cytotoxic at high doses), and Alternariol/Altertoxins acts indirectly via AhR/CYP1 activation rather than direct ER-alpha binding.

Treatment durations across the included studies spanned a wide range, from short-term assays ranging from minutes to a few hours, typically for mechanistic investigations targeting apoptosis and intracellular ROS generation (37, 45), while longer-term studies (5–12 days) were applied, particularly for clonogenic survival, reflecting chronic exposure scenarios (28, 63). Regarding the exposure concentrations, for highly potent estrogenic toxins, the concentrations range from picomolar to nanomolar levels (51, 62) to micromolar for evaluating cytotoxic and pro-apoptotic toxins (43, 48). The full methodological characteristics, particular exposure concentration ranges and the primary phenotypic results of these studies are comprehensively summarized in Supplementary Tables S3, S4.

### 3.3 Estrogenic potency and proliferative dynamics of zearalenone

The mitogenic potential of zearalenone (ZEN) and its structural metabolites is a common finding in the reviewed literature, with studies primarily conducted in estrogen-responsive cell line models, specifically MCF-7 and T47D. Here, the ZEN acts as a potent estrogen agonist, functionally mimicking physiological estrogen exposure to stimulate cell proliferation at environmentally relevant nanomolar concentrations. Quantitative assessments demonstrated that ZEN exposure stimulates cellular proliferation, enhancing the expression of both ERα and ERβ, and promoting cell-cycle progression, where it increased the S-phase population roughly around 50% at 24 h, with an eight-fold rise in cyclin B1-associated kinase activity (29, 36, 46, 50). Several studies demonstrated synergistic activity with estradiol, confirming their tumor-promoting potential, for instance, prior studies demonstrated that ZEN increased the viable cell numbers by +29.2% at 2 nM, +154.5% at 32 nM, and +190.4% at 96 nM, while simultaneously shifting cells from G1 into S phase (S-phase increased roughly from 20% to 40%) (46).

Among the ZEN metabolites, α-zearalenol consistently exhibited the strongest proliferative potency. Prior investigations (36, 42) demonstrated a proliferative effect (PE) of 2.6 relative to untreated controls and a relative proliferative potency (RPP) of 7 relative to 17β-estradiol (E2). RPP defined as the ratio of the half-maximal effective concentration (EC_50_) of the reference estrogen 17β-estradiol (E_2_) to that of the tested mycotoxin derivative. Under this normalization scale, 17β-estradiol (E_2_) is assigned a baseline reference value of 1.0. In contrast, hydrolysed metabolites (HZEN, DHZEN) show negligible estrogenicity. This stark difference highlights that specific structural determinants, specifically an intact lactone ring, are required for ER-mediated proliferation (28, 39). Beyond direct receptor agonism, ZEN promotes tumor survival by activating complex signal transduction networks, including Ras/Erk cascade, cyclin D1, c-myc, and the critical upregulation of anti-apoptotic Bcl-2 proteins (29, 46, 64).

### 3.4 Cytotoxic, apoptotic and genotoxic mycotoxins in breast cancer models

In contrast to the proliferative phenotype induced by mycoestrogens, several mycotoxins demonstrated antiproliferative and pro-apoptotic effects, particularly in receptor-negative (TNBC) cancer cell line models that are inherently resistant to hormone-driven growth. Aflatoxin B1(AFB1) induces aggressive, dose-dependent cellular destruction. Exposure in MCF-7 cells precipitated a viability collapse from 91.7% at 1.2 μg/mL to 31.1% at 9.6 μg/mL, driving apoptosis rates upto 46.4% (59). Targeted cytotoxicity was highly evident among structurally diverse toxins. Gliotoxin suppressed proliferation across six breast cancer cell lines with IC50 values varying from 38 to 985 nM (44). Similarly, Verrucarin A induced dose-dependent apoptosis, with reported IC50 values of 0.41 and 0.29 μM, accompanied by mitochondrial membrane potential loss and ROS generation (37, 53). Among the mycotoxins evaluated, Brefeldin A showed high potency in TNBC cells, with an EC50 of 0.016 μg/mL in MDA-MB-231. Its function seems to be mechanistically different, mainly inhibiting clonogenic activity and causing anoikis (detachment-induced apoptosis) (63). Additionally, Wortmannin significantly increased apoptotic rates from 1.75% at 1 nM to ~25.3% at 50 nM *in vitro* by blocking the PI3K/Akt and NF- κB pathways (47). Upon evaluating miRNA and proteomic changes, (55) demonstrated that a 24-h Wortmannin treatment in BT-20 cells significantly modulated non-coding RNA networks and epithelial-mesenchymal transition markers. Wortmannin suppressed oncogenic microRNAs (including miR-21, miR-193a-5p, and miR-542-5p) while upregulating tumor-suppressive species such as miR-19a/b, miR-22, and miR-29b/c. At the proteomic level, Western blot analysis confirmed a concomitant downregulation of the mesenchymal marker Vimentin and an upregulation of the metastasis suppressor NME1 (NM23-H1), pointing to an *in vitro* suppression of metastatic potential (55).

### 3.5 Modulation of oncogenic signaling and tumor microenvironment dynamics

The research emphasizes that mycotoxins influence cancer progression through multifaceted mechanisms apart from direct cytotoxicity or hormonal stimulation. One important secondary mechanism is metabolic disruption, for example, Alternariol and altertoxins increase CYP1 enzymes and AhR signaling (synergistically reaching ~190% of control levels) (33, 60). Malignant transformation is indirectly catalyzed by this change in endogenous steroid metabolism toward genotoxic products. Acute intracellular stress is a prerequisite for other toxins. Fusaric acid (500 μM) causes G2/M cell cycle arrest and severe endoplasmic reticulum (ER) stress (48). Similarly, a structural analog of ZEA, (5Z)-7-Oxozeaenol causes G1 arrest and ROS generation in about 64% of treated MDA-MB-231 cells (65).

Additionally, the *in vitro* model has a significant impact on apparent toxicity. Compared to conventional 2D monolayers, the intricate microenvironment of 3D spheroids significantly increases resistance to mycotoxins such as Sterigmatocystin (58). Furthermore, cytotoxicity is selective; for example, at identical doses, the response in non-tumorigenic MCF-10A mammary cells contrasts starkly with a 46.4% impact in malignant MCF-7 cells (59). This clear contrast draws attention to how complicated the mycotoxins are. They exhibit selective, pathway-specific cytotoxicity against established malignancies at higher thresholds, while at lower doses they function as environmental cancer promoters in hormone-responsive tissues.

### 3.6 Quality assessment and risk of bias

The methodological quality of the 41 included studies was evaluated utilizing the adapted QUIN tool for *in vitro* studies (27). Overall, none of the studies (0%) were categorized as having a low risk of bias. The vast majority, 95.1% (*n* = 39), demonstrated a medium risk of bias, while the remaining 4.9% (*n* = 2) showed a high risk. Evaluation across the core domains revealed that the primary literature consistently excelled in providing a detailed explanation of the methodology, clear aims and objectives, precise methods of outcome measurement, and transparent presentation of results. Conversely, the most frequent methodological deficiencies were observed in operator and outcome assessor details (indicating a widespread lack of blinding), randomization, and detailed explanations of sample size collection, which were largely absent across the evaluated studies (38, 39, 46, 58–65) (Table 2). All reviewer discrepancies were successfully resolved through mutual consensus, confirming the integrity of the experimental data synthesized in this review.

**Table 2:** Risk of bias evaluation of included studies.

References	Clearly stated aims/objectives	Detailed explanation of sample size collection	Detailed explanation of sampling technique	Details of comparison group	Detailed explanation of methodology	Operator details	Randomization	Methods of outcome measurement	Outcome assessordetails	Statistical analysis	Presentation ofresults	Total score	Final %	Overall risk
Martin et al. (62)	2	0	1	2	2	0	0	2	0	0	2	11	50.0%	Medium
Van Aswegen et al. (44)	2	0	1	2	2	0	0	2	0	0	2	11	50.0%	Medium
Makela et al. (51)	2	0	1	2	2	0	0	2	0	2	2	13	59.1%	Medium
Ahamed et al. (29)	2	0	1	2	2	0	0	2	0	0	2	11	50.0%	Medium
Vigushin et al. (45)	2	0	2	2	2	0	1	2	0	2	2	15	68.2%	Medium
Z. Yu et al. (46)	2	0	0	2	2	0	0	2	0	2	2	12	54.5%	Medium
Minervini et al. (36)	2	0	2	2	2	0	0	2	0	2	2	14	63.6%	Medium
Khosrokhavar et al. (50)	2	0	2	2	2	0	0	2	0	2	2	14	63.6%	Medium
Parveen et al. (39)	2	0	1	2	2	0	0	2	0	2	2	13	59.1%	Medium
Wang et al. (71)	2	0	1	2	2	0	0	2	0	2	2	13	59.1%	Medium
Sondergaard et al. (41)	2	0	0	2	2	0	0	2	0	2	2	12	54.5%	Medium
Yun et al. (47)	2	0	1	2	2	0	0	2	0	2	2	13	59.1%	Medium
Acuña et al. (65)	2	0	1	2	2	0	0	2	0	0	2	11	50.0%	Medium
Rudolph et al. (69)	2	0	2	2	2	0	0	2	0	2	2	14	63.6%	Medium
Jayasooriya et al. (53)	2	0	2	2	2	0	0	2	0	2	2	14	63.6%	Medium
Palanivel et al. (37)	2	0	2	2	2	0	0	2	0	2	2	14	63.6%	Medium
Kaya et al. (34)	2	0	0	1	2	0	0	2	0	2	2	11	50.0%	Medium
Tseng et al. (63)	1	0	2	1	2	0	0	2	0	2	2	12	54.5%	Medium
Parandin et al. (38)	2	1	1	1	2	0	0	2	0	2	2	13	59.1%	Medium
Dellafiora et al. (61)	2	0	1	1	2	0	0	2	0	0	2	10	45.5%	High
Li et al. (55)	2	0	2	2	2	0	0	2	0	2	2	14	63.6%	Medium
Chang et al. (49)	2	0	2	2	2	0	0	2	0	2	2	14	63.6%	Medium
Hessenberger et al. (32)	2	0	2	2	2	0	0	2	0	2	2	14	63.6%	Medium
Qi et al. (40)	2	0	2	0	2	0	0	2	0	2	2	12	54.5%	Medium
Yip et al. (3)	2	0	2	1	2	0	0	2	0	2	2	13	59.1%	Medium
Nittoli et al. (56)	2	0	0	0	2	0	0	2	0	2	2	10	45.5%	High
Tatay et al. (42)	2	0	1	1	2	0	0	2	0	2	2	12	54.5%	Medium
Fruhauf et al. (28)	2	0	1	2	2	0	0	2	0	2	2	13	59.1%	Medium
Lecomte et al. (35)	2	0	1	0	2	0	0	2	0	2	2	11	50.0%	Medium
Hohenbichler et al. (33)	2	0	2	1	2	0	0	2	0	2	2	13	59.1%	Medium
Zhao et al. (59)	2	0	2	0	2	0	0	2	0	2	2	12	54.5%	Medium
Kowalska et al. (60)	2	0	1	1	2	0	0	2	0	2	2	12	54.5%	Medium
Turkmen et al. (43)	2	0	2	0	2	0	0	2	0	2	2	12	54.5%	Medium
Adam et al. (59)	2	0	2	2	2	0	0	2	0	2	2	14	63.6%	Medium
Zhang et al. (49)	2	0	2	2	2	0	0	2	0	2	2	14	63.6%	Medium
Malekinejad et al. (64)	2	0	2	1	2	0	0	2	0	2	2	13	59.1%	Medium
Asnaashari et al. (31)	2	0	2	2	2	0	0	2	0	2	2	14	63.6%	Medium
Aljazzar et al. (30)	2	0	2	1	2	0	0	2	0	2	2	13	59.1%	Medium
Juan-García et al. (54)	2	0	2	1	2	0	0	2	0	2	2	13	59.1%	Medium
Zingales et al. (58)	2	0	2	2	2	0	0	2	0	2	2	14	63.6%	Medium
Xu et al. (52)	2	0	2	2	2	0	0	2	0	2	2	14	63.6%	Medium

Scoring criteria: Adequately specified = 2, Inadequately specified = 1, Not specified = 0, Not applicable. Number of applicable criteria for all the included studies were 11. Thus, the final score = Total score × 100 / (2 × number of applicable criteria). Risk categories: >70% = Low risk; 50–70% = Medium risk; < 50% = High risk.

## 4 Discussion

Data from *in vitro* investigations on human breast cancer models suggest that mycotoxin has a non-uniform effect that may vary significantly depending on the type of cell and receptor status. Mycotoxin cannot be treated as a single pharmacological entity, as evidenced by the range of biological reactions seen, such as cell growth, programmed cell death, cell cycle disruption, oxidative stress, DNA damage, modulation of oncogenic signaling pathways, and interaction with hormone receptors. Rather, they constitute a structurally and mechanistically varied group of fungal metabolites that require substantial scientific and public health attention due to their possible significance in the biology of breast cancer (14, 15).

Despite the established biological significance of estrogenic mycotoxins, particularly zearalenone (ZEA) and its derivatives, in hormone-sensitive breast cancer cell line models, a significant proportion of the analyzed literature focused on estrogen-receptor expressing ER+ lineages, such as MCF-7. These results provide a translational link between *in vitro* mechanistic evidence and human disease burden (17, 66). The widespread presence of ZEA and related mycoestrogens in staple foods is particularly prevalent in regions reliant on maize- and cereal-based diets (21).

### 4.1 Zearalenone and derivatives: predominant estrogenic potency and mechanistic basis

Among all the mycotoxins assessed, zearalenone (ZEA), along with its primary metabolites α-ZOL, β-ZOL, and zearalenone, demonstrated the most consistent and mechanistically well-defined mitogenic impacts on estrogen-sensitive breast carcinoma models. The estrogenic activity of ZEA results from its high-affinity binding capacity to estrogen receptors alpha (ERα) and beta (ERβ), with an affinity that, in the case of α-ZOL, approaches that of 17β-estradiol (E2) itself (29, 36). Once bound to ERα, ZEA and α-ZOL induce the transcriptional activation of estrogen-regulated genes, specifically upregulating cyclin D1, c-Myc oncogene, PgR expression, driving MCF-7 and T47D cells through the G1/S-phase transition at concentrations as low as 2nM, concentrations achievable through normal dietary exposure (29, 46).

Quantitatively, previous research demonstrated that ZEA increased viable MCF-7 cell numbers by +29.2% at 2 nM, +154.5% at 32 nM, and +190.4% at 96 nM, while shifting cells from G1 into S phase (from 20% to 40%) (46). ZEA increased the S-phase population from 10% to 50% at 24 h, with an 8-fold rise in cyclin B1-associated kinase activity. Among ZEA metabolites, alpha-zearalenol demonstrated the strongest proliferative potency, with an RPP of 7 relative to 17β-estradiol (29) substantially exceeding the potency of parent ZEA, which is not directly captured by the RPP metric itself. This superior potency results from stereospecific reduction of the C-6 keto group, producing a steric orientation more compatible with the antagonist conformation of ER-alpha (36). Conversely, hydrolysed metabolites HZEN and DHZEN showed negligible estrogenicity, confirming that intact lactone ring structure and specific stereochemical configuration are prerequisites for ER binding (28).

Beyond simply promoting cell growth, ZEA also activates multiple cancer-related signal transduction networks, such as overexpression of the Ras/Raf/ERK signaling pathway, which promotes cellular longevity and resistance to apoptosis, while Bcl-2 upregulation suppresses mitochondrial-mediated apoptotic cell death (29, 52, 64). ZEA additionally modulates the PI3K/Akt/mTOR axis under ER-dependent conditions, promoting metabolic reprogramming and resistance to anti-mitogenic signals (46). This multi-pathway oncogenic activation, including the maintenance of mitogenic signaling, evading growth suppressors, and resisting apoptotic triggers, makes ZEA a genuine tumor-promoting endocrine disruptor (20).

Furthermore, ZEA and its metabolites are among the most globally prevalent food contaminants, detected in maize, wheat, barley, and meat products (14) enabling the chronic low-dose estrogenic stimulation that parallels known hormonal risk factors for breast cancer, including early menarche and prolonged hormone use (66).

### 4.2 Comparative effects of other mycotoxins and cell line sensitivity

While ZEA dominated estrogenic proliferative effects, other mycotoxins demonstrated distinct biological impacts. AFB1 exerted predominantly cytotoxic and genotoxic effects: in MCF-7 cells, it reduced cellular viability from 91.7% at 1.2 μg/mL to 31.1% at 9.6 μg/mL with apoptosis rates up to 46%, via CYP450-mediated bioactivation to AFB1-8, 9-epoxide intermediates, which form genomic adducts. This biochemical interaction triggers GC-to-TA transversion mutations capable of inactivating TP53 (17, 67). OTA demonstrated cytotoxicity with IC50 ~3 μM at 24 h via ROS-mediated oxidative stress and intrinsic apoptosis (54, 68). Notably, Zingales et al. demonstrated that 3D spheroid cultures (58) were substantially more resistant to OTA than 2D monolayer cultures, underscoring the need for physiologically complex *in vitro* models. Beauvericin and enniatin B, functioning as ionophoric compounds, demonstrated IC_50_ values of 1.8 μM and 0.15 μm (54), respectively. Importantly, Yip et al. showed that combined low-dose ZEA and AFB1 promoted greater cell cycle progression in MCF-7 cells than either compound alone, highlighting the biological plausibility of synergistic mycotoxin interactions under realistic dietary co-exposure conditions (3)

Alternariol and altertoxins activated the aryl hydrocarbon receptor (AhR) and induced CYP1 enzyme activity to around 190% of controls. This is mechanistically significant as CYP1B1, an AhR target gene, converts estradiol to the genotoxic, catecholestrogens 4-hydroxyestradiol, representing an indirect pathway through which non-estrogenic mycotoxins may promote estrogenic carcinogenesis (33, 60). Wortmannin inhibited PI3K/Akt and NF-κB signaling, enhancing apoptosis from 1.75% at 1 nM to 25.3% at 50 nM (47), beyond direct cell viability reduction, Wortmannin attenuate aggressive cell line phenotypes through non-coding RNA dysregulation and EMT suppression. By suppressing Vimentin and enhancing NME1 expression in BT-20 cells, Wortmannin blocks key cellular features associated with cell motility and invasive potential. These observations, combined with targeted inhibition of the PI3K/Akt pathway and pro-apoptotic induction reported across other cell line studies, underscore its broader role in suppressing metastatic signaling cascades *in vitro* (55).

A clinically meaningful dichotomy emerged across breast cancer subtypes. Estrogen-responsive (ER+) lines such as T47D, MCF-7, ZR-75-1 exhibited proliferative responses to estrogenic mycotoxins at nanomolar concentrations via intact ERα/ERβ signaling, while TNBC cell lines like MDA-MB-468, MDA-MB-231, BT-549, BT-20 were largely resistant to ER-mediated proliferative effects but vulnerable to cytotoxic compounds including brefeldin A (EC50 ~0.016 μg/mL in MDA-MB-231) (64), gliotoxin (IC_50_ 38–985 nM across six lines) (45), and verrucarin A (IC50 0.29–0.41 μM) (37). Oxozeaenol, a structural analog of ZEA, induced ROS accumulation and G1 arrest in ~64% of MDA-MB-231 cells via TAK1 inhibition rather than ER agonism (65), and curvularins with galiellalactone suppressed TGF-β-induced vasculogenic mimicry in TNBC cells (69). Non-tumorigenic MCF-10A cells consistently showed reduced sensitivity relative to tumor lines, with apoptosis rates of 40.8% vs. 46.4% in MCF-7 under AFB1 exposure, confirming selective tumor cell vulnerability. ZEA exhibits a biphasic hermetic response, promoting cell growth at nanomolar levels while inducing cytotoxicity at elevated concentrations. This pattern suggests that carcinogenic risks from increased, low-dose dietary intake may be significantly underestimated by typical IC50-derived risk assessments (70).

### 4.3 Epidemiological gaps and limitations

The epidemiological literature that directly links mycotoxin exposure to human breast cancer is still conflicting, despite having strong *in vitro* mechanistic evidence. Claeys et al. (14) has identified that only a small number of case control studies have examined the ZEA biomarker in relation to breast cancer; these studies produced inconsistent results, which are probably due to variations in biomarker selection, assay sensitivity and dietary assessment methods. The major focus of human mycotoxin-carcinogenesis research on hepatocellular carcinoma has left hormone-dependent cancer substantially understudied. This gap is particularly concerning for populations in developing regions where ZEA-contaminated staple foods constitute the dietary foundation and breast cancer incidence is rising alongside limited access to early detection and targeted therapies (7, 17). The convergence of *in vitro* mechanistic evidence with available biomonitoring data provides, however, a scientifically credible framework for aetiological investigations confirmed that xenoestrogens, including ZEA, activate the same transcriptional programmes that underpin established hormonal risk factors for breast cancer (20).

Key methodological limitations include the predominant use of 2D monolayer culture systems. As highlighted by Zingales et al. (58), 3D spheroid architectures demonstrate profound resistance to mycotoxin-induced cytotoxicity compared to conventional 2D models. Because 2D monolayers lack the complex spatial architecture and stromal interactions of the native tumor microenvironment, they likely overestimate the pharmacological and cytotoxic sensitivity of these cells. Consequently, current *in vitro* thresholds may not accurately reflect physiological tissue responses. Additional limitations include wide concentration ranges that complicate cross-study comparisons, the near-exclusive examination of individual mycotoxins rather than realistic co-exposure mixtures, and a paucity of epigenetic endpoint studies despite a strong mechanistic rationale (67, 68).

A formal risk of bias assessment of the included literature was conducted using the QUIN tool (27), however, a key limitation remains the field-wide lack of a universally accepted, consensus-validated quality appraisal framework designed specifically for basic cell line and molecular oncology research (26). The strength of the synthesized mechanistic evidence is inherently tied to the methodological rigor of the primary *in vitro* literature. Our quality assessment revealed that while the overarching experimental designs and outcome measurements were robust, there were ubiquitous omissions in operator blinding, outcome assessor blinding, randomization, and formal sample size calculations. It must be acknowledged that the adapted appraisal tool (27) derives certain domain criteria from clinical or applied *in vitro* settings; thus, expectations regarding a priori sample size calculations and strict blinding may not fully align with standard exploratory cell culture practices (Table 2). Despite this contextual caveat, these pervasive methodological reporting gaps introduce a potential risk of bias, warranting a cautious interpretation of the literature, particularly when evaluating marginal cytotoxic thresholds.

Future research priorities should include: (i) enhanced regulatory surveillance of ZEA in human food supplies; (ii) prospective epidemiological cohort studies with validated ZEA/metabolite biomarkers; (iii) 3D organoid and co-exposure experimental paradigms; and (iv) epigenetic endpoint characterization in breast epithelial models to establish the full carcinogenic significance of chronic dietary mycotoxin exposure.

## 5 Conclusion

This systematic review consolidates fragmented *in vitro* literature to establish a strong mechanistic framework regarding mycotoxin activity in breast cancer models. While the current evidence does not establish direct causality for human breast cancer, it robustly demonstrates biological plausibility. Specifically, ZEA and α-ZOL emerge as profound mechanistic drivers of hormone-sensitive proliferation due to their high-affinity ERα/ERβ binding capacity, dose-dependent mitogenesis at environmentally relevant nanomolar concentrations, multi-pathway oncogenic signaling activation (Ras/ERK, Bcl-2, cyclin D1, c-myc, PI3K/Akt/mTOR), structural metabolic determinants of superior α-ZOL potency over parent ZEA, and epidemiological biomarker evidence linking ZEA metabolites to breast cancer. Other mycotoxins, including AFB1, OTA, gliotoxin, brefeldin A, verrucarin A, beauvericin, enniatin B, alternariol, and wortmannin, contribute to a broader mechanistic picture through genotoxic, cytotoxic, AhR-activating, and kinase-inhibitory mechanisms. Furthermore, while these findings present a robust mechanistic framework, the prevalent lack of blinding and formal sample size calculations across the existing *in vitro* literature highlights a medium risk of bias, necessitating cautious interpretation of marginal cytotoxic thresholds.

## Data Availability

The original contributions presented in the study are included in the article/Supplementary material, further inquiries can be directed to the corresponding author.
