# Supplementary material for: Estrogenic, cytotoxic, proliferative effects of mycotoxins in human breast cancer cell line models: a systematic review
Source: Front Public Health. 2026 Oct 30;14:1950615. doi: 10.3389/fpubh.2026.1950615 (PMC13759722; doi:10.3389/fpubh.2026.1950615)
Supplement: Supplementary file 1 [file Table_1.docx]

**Supplementary Table S4.** Overview of biological responses and primary phenotypic outcomes induced by mycotoxins in breast cell models.

| **S.no** | **Study (Author, Year, references)** | **Mycotoxin Evaluated** | **Primary Phenotypic Outcomes (Viability & Cytotoxicity)** | **Estrogenic Activity & Receptor Modulation** | **Identified Mechanistic Pathways** | **Control Medium** |
| --- | --- | --- | --- | --- | --- | --- |
| 1 | Martin et al., 1978 [62] | Zearalenone (ZEN), Zearalenol LMP/HMP | Enhanced log-phase cellular growth above control baselines. | High ER binding affinity: Zearalenol HMP (1 nM) > ZEN (6 nM). | Modulation of Sex Hormone-Binding Globulin (SHBG). | EME Medium |
| 2 | Van Aswegen et al., 1985 [44] | Phomopsin | Significant cytotoxicity at 1 µM. Total cell numbers markedly reduced. | None reported. | Severe inhibition of DNA replication (thymidine incorporation down to 41%) and protein synthesis (glycine down to 36%). | Untreated |
| 3 | Makela et al., 1994 [51] | Zearalenone | Increased cell viability and synergistic proliferation when combined with estradiol. | Full estrogenic agonist activity at low concentrations. | ER-dependent gene activation (confirmed via pS2 expression). | DMEM + 5% FBS |
| 4 | Ahamed et al., 2001 [29] | Zearalenone | Dose-dependent inhibition of apoptosis. G1→S phase transition accelerated (G1 reduced 66%→36%; S increased 20%→40%). | Strong estrogenic tumor-promoting effect in ER+ breast cancer models. | Upregulation of anti-apoptotic Bcl-2; downregulation of pro-apoptotic Bax. | Ethanol |
| 5 | Vigushin et al., 2004 [45] | Gliotoxin | Broad-spectrum antiproliferative activity across 6 cell lines. IC₅₀ = 38–985 nM. | None reported. | Dual inhibition of tumor signaling enzymes FTase (80 µM) and GGTase I (17 µM). | DMSO |
| 6 | Z. Yu et al., 2005 [46] | Zearalenone | Low doses (1–10 nM) caused ~2-fold growth stimulation. High doses (>1 µM) caused toxicity. | Strictly ER-dependent; no effect on ER-negative MDA-MB-231 cells. | Estrogen receptor-mediated cell cycle progression. | E2 Control |
| 7 | Minervini et al., 2005 [36] | ZEN & Derivatives | Cell proliferation induced at low nM concentrations across all tested compounds. | α-ZOL exhibited the strongest proliferative effect (PE = 2.6, RPP = 7). β-ZOL was weakest. | Target gene activation correlates strongly with 17β-estradiol (R=0.93–0.96). | 10 nM E2 |
| 8 | Khosrokhavar et al., 2009 [50] | Zearalenone, α-ZOL | Stimulated cell proliferation and increased overall cell viability. | Upregulated ERα and ERβ mRNA expression (p < 0.05). | Activation of estrogenic signaling; increased Bcl-2/Bax ratio promoting survival. | Untreated |
| 9 | Parveen et al., 2009 [39] | ZEN & Analogues | Induced proliferation at low nM concentrations. | α-ZOL demonstrated strongest estrogenic potency. | cDNA microarray confirmed activation of identical target genes as E2. | 0.5% Ethanol |
| 10 | Wang et al., 2010 [71] | Zearalenone | Promoted cell proliferation and viability. | ER-mediated growth stimulation. | Activation of cell-cycle progression pathways. | Untreated |
| 11 | Sondergaard et al., 2012 [41] | Fusarin C | Proliferative at moderate doses (EC₅₀ = 890 nM). Cytotoxic at high doses (IC₅₀ = 46.8 µM). | Demonstrated significant estrogenic activity. | ER transactivation confirmed via CALUX reporter assay. | Ethanol |
| 12 | Yun et al., 2012.[47] | Wortmannin | Dose-dependent inhibition (up to 51% at 50 nM). Apoptosis increased from 1.7% to 25.3%. | None reported. | Suppression of PI3K/Akt and NF-κB p65 expression. | DMSO |
| 13 | Acuña et al., 2012 [65] | (5Z)-7-Oxozeaenol | Dose-dependent cytotoxicity. Induced G1-phase cell cycle arrest in ~64% of TNBC cells. | None reported. | Severe ROS accumulation; concentration-dependent inhibition of NF-κB (p65, p50). | Untreated |
| 14 | Rudolph et al., 2013 [69] | Galiellalactone, Curvularins | Suppressed cellular proliferation, migration, and invasive capacity. | None reported. | Inhibition of TGF-β-induced vasculogenic mimicry. | Untreated |
| 15 | Jayasooriya et al., 2013 [53] | Verrucarin A | Dose-dependent apoptosis; synergistically enhanced TNF-α-induced cell death. | None reported. | Overexpression of NF-κB-mediated Fas apoptotic pathways. | Untreated |
| 16 | Palanivel et al., 2014 [37] | Verrucarin A | Strong cytotoxicity (IC₅₀ = 0.41 and 0.29 µM). | None reported. | Loss of mitochondrial Δψm; ROS generation; p38 MAPK activation. | Untreated |
| 17 | Kaya et al., 2014 [34] | α- and β-Amanitin | α-Amanitin highly potent (LD₅₀ = 1 µg/mL). Viability reduced by ~50%. | None reported. | Rapid inhibition of RNA polymerase II and total cellular protein synthesis. | Untreated |
| 18 | Tseng et al., 2014 [63] | Brefeldin A | Exceptionally potent in TNBC (EC₅₀ ≈ 0.016 µg/mL). Induced anoikis and suppressed clonogenicity. | None reported. | Extreme ER-stress activation (↑IRE1α, PERK, CHOP); PARP and caspase cleavage. | DMEM + 10% FCS |
| 19 | Parandin et al., 2015 [38] | Zearalenone, α-ZOL | Increased viability in MCF-7 cells at 24/48h (p < 0.05). No effect on ER- MDA-MB-468 cells. | α-ZOL demonstrated greater proliferative activity than parent ZEN. | ER-dependent mitogenic signaling. | Untreated |
| 20 | Dellafiora et al., 2016 [61] | ZEN-14-glucoside | No direct cytotoxicity observed up to 1 mM (masked toxin). | MCF-7 cells enzymatically converted the glucoside into active estrogenic ZEN, α-ZOL, and β-ZOL over 2–6h. | Enzymatic unmasking restores ER-binding capacity and tumor-promoting activity. | Untreated |
| 21 | Li et al., 2016 [55] | Wortmannin | Not reported. | None reported. | Targeted suppression of signal transduction pathways. Significant downregulation of the mesenchymal marker protein Vimentin. Marked upregulation of NME1 (NM23-H1) protein expression. | Untreated |
| 22 | Chang et al., 2016 [49] | Cytochalasin B | High sensitivity in ZR-75-1 cells (IC₅₀ ≈ 12.5 µM, 98.2% death). No toxicity in normal MCF-10A cells. | None reported. | Intracellular Ca²⁺ elevation; severe mitochondrial dysfunction, and cell cycle arrest. | Untreated |
| 23 | Hessenberger et al., 2017 [32] | Zearalenone | Significant increase in cell proliferation and viability (p < 0.001). | Synergistic proliferative activity when combined with phytoestrogens. | ER-mediated DNA synthesis and cell cycle acceleration. | Untreated |
| 24 | Qi et al., 2017 [40] | Ochratoxin A | Growth inhibition dependent on transporter expression (IC₅₀ ≈ 12.8–16.4 µM). | None reported. | pH-dependent efflux/uptake modulated by mBcrp, hBCRP, and MRP2 transporters. | DMEM |
| 25 | Yip et al., 2017 [3] | ZEN, Aflatoxin B1 | AFB1 induced cytotoxicity and successfully suppressed ZEN-induced proliferation in co-exposure. | ZEN promoted proliferation; AFB1 antagonised this effect via cytotoxicity. | Antagonistic biological interplay between estrogenic and genotoxic mechanisms. | Untreated |
| 26 | Nittoli et al., 2018 [56] | α-Zearalenol | Accelerated cell cycle progression (increased S-phase and G2/M phase). | Strong selective estrogenic effect in ERP lines; no effect in ERN lines. | Altered metabolic profiling (enhanced protein & lipid metabolism). | Untreated |
| 27 | Tatay et al., 2018 [42] | ZEN, α-ZOL, β-ZOL | Proliferative Effect (PE) ranged 1.05–1.62. High doses (18.75–25 µM) resulted in cell death. | α-ZOL demonstrated the highest relative estrogenic potency due to superior ER affinity. | ER-dependent transcriptional activation. | Negative Control |
| 28 | Fruhauf et al., 2019 [28] | ZEN, HZEN, DHZEN | ZEN increased proliferation up to 226%. HZEN/DHZEN showed no proliferative response. | HZEN and DHZEN were ~50 to 10,000× less estrogenic than intact ZEN. | Reporter gene assay confirmed loss of lactone ring destroys ER transactivation. | DMSO |
| 29 | Lecomte et al., 2019 [35] | ZEN, Apigenin | ZEN induced proliferation to ~80% of the native Estradiol (E2) effect. | Full ERα agonist activity initiated at 1 nM; maximum response at 10 nM. | ER-mediated induction of the Progesterone Receptor (PgR) gene. | E2 Control |
| 30 | Hohenbichler et al., 2020 [33] | Alternariol, ATX-I/II | Largely non-cytotoxic at tested doses (0.01–0.1 µM). | Indirect estrogenic effect via disruption of estrogen metabolism. | Aryl Hydrocarbon Receptor (AhR) activation; EROD/CYP1 activity increased ~190%. | DMSO |
| 31 | Zhao et al., 2021 [59] | 10,11-Dehydrocurvularin | Dose- and time-dependent inhibition of proliferation; increased Annexin V/PI-positive cells. | None reported. | Induction of apoptosis via cleaved PARP and caspase-3 activation. | Untreated |
| 32 | Kowalska et al., 2021 [60] | Alternariol (AOH) | Dose/time-dependent viability drop (IC₅₀ = 23.97 µM). High doses caused oxidative stress (~2-fold). | Low concentrations (0.1 µM) generated estrogen-like proliferative effects. | Dual action: Estrogenic at low doses; genotoxic (DNA damage, G2/M arrest) at high doses. | Untreated |
| 33 | Turkmen et al., 2021 [43] | Patulin | IC₅₀ ≈ 25 µM in MCF-7 cells. Highly selective: at 100 µM, cancer viability fell to 12% vs 36% in normal cells. | None reported. | Time-dependent apoptotic cell death. | Untreated |
| 34 | Adam et al., 2022 [59] | Aflatoxin B1 | MCF-7 viability collapsed to 31.1% (at 9.6 µg/mL). Apoptosis reached 46.4%. | None reported. | Severe ROS modulation; altered oncogene and tumor suppressor expression. | Untreated |
| 35 | Zhang et al., 2023 [49] | Fusaric Acid | 100 µM reduced colony formation. 500 µM triggered cell cycle arrest. | None reported. | Endoplasmic Reticulum (ER) stress pathway activation; G2/M phase arrest. | Untreated |
| 36 | Malekinejad et al., 2023 [64] | ZEN, α-ZEL, β-ZEL | Increased cell proliferation (10–40%). | Stimulated proliferation via ERα signalling. | Shifted estrogen metabolism toward carcinogenic 4-OHE2 via CYP1A1/CYP1B1. | DMSO |
| 37 | Asnaashari et al., 2023 [31] | Zearalenone | Biphasic: Low doses increased migration/EMT; high doses triggered cytotoxicity. | Low doses strictly mimicked E2, driving stemness in ER+ cells. | EMT modulation (decreased E-cadherin, increased vimentin at low doses). | Untreated |
| 38 | Aljazzar et al., 2023 [30] | Aflatoxin B1 | Dose-dependent cytotoxicity (100–1000 nM). Negative correlation with ROS generation. | None reported. | Inflammatory response (↑COX-2, TNF-α); upregulation of CYP1A1, MDR1, MRP2. | Untreated |
| 39 | Juan-García et al., 2024 [54] | BEA, ENN B, OTA | Potent cytotoxicity. 24h IC₅₀ values: ENN B (0.15 µM) > BEA (1.8 µM) > OTA (3 µM). | None reported. | Ionophoric disruption of cellular gradients. | Untreated |
| 40 | Zingales et al., 2024 [58] | STE, OTA, PAT | Patulin is most potent. IC₅₀ decreased over time (24→72h). | None reported. | 3D spheroid architectures demonstrated profound resistance compared to 2D models. | Untreated |
| 41 | Xu et al., 2025 [52] | Zearalenone | Time and dose-dependent cellular proliferation observed. | ER-mediated mitogenic effect. | Upregulation of downstream oncogenic transcription factors. | Untreated |

Summary of the primary phenotypic responses observed across the evaluated literature were provided, broadly categorizing the effects of each mycotoxin into proliferative, cytotoxic, or estrogenic activities, alongside a brief outline of the physiological outcomes in exposed cell cultures

BEA= Beauvericin; EME Medium = Eagle's Minimum Essential Medium; ENN= Enniatin; OTA= Ochratoxin A; STE= Sterigmatocystin; PAT=Patulin; ZEN= Zearalenone; α-ZOL=alpha-Zearanolol; LD₅₀=Lethal Dose,50%; IC₅₀= Half maximum inhibitory concentration; EC₅₀= Half maximal effective concentration.
