# Supplementary material for: Estrogenic, cytotoxic, proliferative effects of mycotoxins in human breast cancer cell line models: a systematic review
Source: Front Public Health. 2026 Oct 30;14:1950615. doi: 10.3389/fpubh.2026.1950615 (PMC13759722; doi:10.3389/fpubh.2026.1950615)
Supplement: Supplementary file 2 [file Table_2.docx]

**Supplementary table S2: Search strategy of all databases**

| **Search number** | **Query** | **No. of articles** |
| --- | --- | --- |
| **MEDLINE (PubMed)** | | |
| #1 | ((((((mycotoxin[MeSH Terms]) OR (fungal toxins[MeSH Terms])) OR (aflatoxin[MeSH Terms])) OR (aflatoxin b[MeSH Terms])) OR (aflatoxin b 1[MeSH Terms])) OR (ochratoxin[MeSH Terms])) | 53678 |
| #2 | ((((((breast neoplasm[MeSH Terms]) OR (breast tumors[MeSH Terms])) OR (breast cancer[MeSH Terms])) OR (breast malignant neoplasm[MeSH Terms])) OR (breast malignant tumor[MeSH Terms])) OR (human mammary carcinoma[MeSH Terms])) | 378768 |
| #3 | ((cell lines[MeSH Terms]) OR (cell line[MeSH Terms])) | 126287 |
| #4 | #1 AND #2 AND #3 | 70 |
| **Embase** | | |
| #1 | 'fungal toxin'/exp OR 'fungal toxin' OR 'fungus toxin'/exp OR 'fungus toxin' OR 'mycotoxin'/exp OR 'mycotoxin' OR 'mycotoxine'/exp OR 'mycotoxine' OR 'mycotoxins'/exp OR 'mycotoxins' OR 'ochratoxin'/exp OR 'ochratoxin' OR 'ochratoxin a'/exp OR 'ochratoxin a' OR 'ochratoxin a1'/exp OR 'ochratoxin a1' OR 'ochratoxin b'/exp OR 'ochratoxin b' OR 'ochratoxin c'/exp OR 'ochratoxin c' OR 'ochratoxine a'/exp OR 'ochratoxine a' OR 'ochratoxins'/exp OR 'ochratoxins' OR 'aflatoxin'/exp OR 'aflatoxin' OR 'aflatoxin 1'/exp OR 'aflatoxin 1' OR 'aflatoxine'/exp OR 'aflatoxine' OR 'aflatoxins'/exp OR 'aflatoxins' OR 'crude aflatoxin'/exp OR 'crude aflatoxin' | 91358 |
| #2 | 'ca breast'/exp OR 'ca breast' OR 'breast cancer'/exp OR 'breast cancer' OR 'breast gland cancer'/exp OR 'breast gland cancer' OR 'breast gland neoplasm'/exp OR 'breast gland neoplasm' OR 'breast malignancies'/exp OR 'breast malignancies' OR 'breast malignancy'/exp OR 'breast malignancy' OR 'breast tumor malignant'/exp OR 'breast tumor malignant' OR 'cancer in the mammary gland'/exp OR 'cancer in the mammary gland' OR 'cancer of the breast'/exp OR 'cancer of the breast' OR 'cancer of the mammary gland'/exp OR 'cancer of the mammary gland' OR 'cancer, breast'/exp OR 'cancer, breast' OR 'malignancies of the breast'/exp OR 'malignancies of the breast' OR 'malignancy of the breast'/exp OR 'malignancy of the breast' OR 'malignant breast neoplasm'/exp OR 'malignant breast neoplasm' OR 'malignant breast tumor'/exp OR 'malignant breast tumor' OR 'malignant neoplasm of the breast'/exp OR 'malignant neoplasm of the breast' OR 'malignant tumor of the breast'/exp OR 'malignant tumor of the breast' OR 'mamma cancer'/exp OR 'mamma cancer' OR 'mammary cancer'/exp OR 'mammary cancer' OR 'mammary gland cancer'/exp OR 'mammary gland cancer' OR 'mammary gland malignancy'/exp OR 'mammary gland malignancy' OR 'mammary malignancies'/exp OR 'mammary malignancies' OR 'mammary malignancy'/exp OR 'mammary malignancy' OR 'bc cell line (breast cancer)'/exp OR 'bc cell line (breast cancer)' OR 'breast cancer cell line'/exp OR 'breast cancer cell line' OR 'breast cancer-derived cell line'/exp OR 'breast cancer-derived cell line' OR 'breast malignancy cell line'/exp OR 'breast malignancy cell line' OR 'breast malignant cell line'/exp OR 'breast malignant cell line' OR 'cancer breast cell line'/exp OR 'cancer breast cell line' OR 'cancerous breast cell line'/exp OR 'cancerous breast cell line' OR 'malignant breast cell line'/exp OR 'malignant breast cell line' OR 'malignant mammary cell line'/exp OR 'malignant mammary cell line' OR 'mammary cancer cell line'/exp OR 'mammary cancer cell line' OR 'mammary gland cancer cell line'/exp OR 'mammary gland cancer cell line' OR 'mammary gland malignant cell line'/exp OR 'mammary gland malignant cell line' | 850768 |
| #3 | 'cell line'/exp OR 'cell line' OR 'cell lines'/exp OR 'cell lines' OR 'line, cell'/exp OR 'line, cell' | 139189 |
| #4 | #1 AND #2 AND #3 | 965 |
| **Web of Science** | | |
| #1 | ( TS=(mycotoxin) OR TS=("fungal toxins") OR TS=(aflatoxin) OR TS=("aflatoxin b") OR TS=("aflatoxin b1") OR TS=(ochratoxin) ) | 44325 |
| #2 | ( TS=("breast neoplasm") OR TS=("breast tumors") OR TS=("breast cancer") OR TS=("breast malignant neoplasm") OR TS=("breast malignant tumor") OR TS=("human mammary carcinoma")) | 599876 |
| #3 | (TS=("cell lines") OR TS=("cell line") ) | 660987 |
| #4 | #1 AND #2 AND #3 | 54 |
| **Scopus** | | |
| #1 | ( TITLE-ABS-KEY ( mycotoxin ) OR TITLE-ABS-KEY ( "fungal toxins" ) OR TITLE-ABS-KEY ( aflatoxin ) OR TITLE-ABS-KEY ( "aflatoxin b" ) OR TITLE-ABS-KEY ( "aflatoxin b1" ) OR TITLE-ABS-KEY ( ochratoxin ) ) | 66325 |
| #2 | ( TITLE-ABS-KEY ( "breast neoplasm" ) OR TITLE-ABS-KEY ( "breast tumors" ) OR TITLE-ABS-KEY ( "breast cancer" ) OR TITLE-ABS-KEY ( "breast malignant neoplasm" ) OR TITLE-ABS-KEY ( "breast malignant tumor" ) OR TITLE-ABS-KEY ( "human mammary carcinoma" ) ) | 698923 |
| #3 | (TITLE-ABS-KEY ( "cell lines" ) OR TITLE-ABS-KEY ( "cell line" ) ) | 161178 |
| #4 | #1 AND #2 AND #3 | 103 |
| **Google scholar** | | |
|  | ("mycotoxin" OR "fungal toxin" OR "aflatoxin" OR "aflatoxin B1" OR "aflatoxin B" OR "ochratoxin") AND ("breast cancer" OR "breast neoplasm" OR "breast tumor" OR "breast carcinoma" OR "human mammary carcinoma" OR "breast malignancy") AND ("cell line" OR "cell lines" OR "in vitro" OR "MCF-7" OR "T47D" OR "MDA-MB-231") | 40 |
