# Supplementary material for: Estrogenic, cytotoxic, proliferative effects of mycotoxins in human breast cancer cell line models: a systematic review
Source: Front Public Health. 2026 Oct 30;14:1950615. doi: 10.3389/fpubh.2026.1950615 (PMC13759722; doi:10.3389/fpubh.2026.1950615)
Supplement: Supplementary file 3 [file Table_3.docx]

**Supplementary Table S3. Characteristics of included in vitro studies evaluating mycotoxin exposure in breast cancer cell lines.**

| **S.no** | **Study (Author, Year, references)** | **Cell Line Model (Receptor Status)** | **Mycotoxin(s) Evaluated** | **Culture Medium** | **Exposure Concentration** | **Analytical Assays** | **Duration** | **Funding Source** |
| --- | --- | --- | --- | --- | --- | --- | --- | --- |
| 1 | Martin et al., 1978 [62] | MCF-7 (ER+) | Zearalenone, Zearalenol LMP, Zearalenol HMP | Eagle's MEM + 5% Calf Serum | 0.02 µM – 0.3 µM | Protamine sulfate assay | 8 days | NIH; American Cancer Society; Welch Foundation |
| 2 | Van Aswegen et al., 1985 [44] | MCF-7 (ER+) | Phomopsin | Eagle's MEM + L-glutamine | 1 µM | Not specified | 24 h | Phi Beta Psi Sorority; South African Medical Council |
| 3 | Makela et al., 1994 [51] | MCF-7, T-47D (ER+) | Zearalenone | DMEM + 5–10% FBS + Insulin | 0.1 nM – 0.1 µM | MTT assay | 24 h | Jahnsson, Turku Univ., & Emil Aaltonen Foundations |
| 4 | Ahamed et al., 2001 [29] | MCF-7 (ER+) | Zearalenone | DMEM + 5% FCS | 0.1 µM | MTT; Flow cytometry; Annexin V | 50 h | NIH / NCI (USA) |
| 5 | Vigushin et al., 2004 [45] | MCF-7, T47D, BT-474, ZR-75-1 (ER+); MDA-MB-231, MDA-MB-435 (TNBC) | Gliotoxin | DMEM + 10% FCS | 1 nM – 10 µM | SRB assay; Prenyltransferase assays | 48 h | Charing Cross Hospital Trustees |
| 6 | Z. Yu et al., 2005 [46] | MCF-7 (ER+) | Zearalenone | DMEM + 5% FCS | 2 nM – 96 nM | MTT; Flow cytometry; Western blot; RT-PCR | 72 h | NSFC (China) |
| 7 | Minervini et al., 2005 [36] | MCF-7 (ER+), MDA-MB-231 (ER-) | Zearalenone & derivatives | RPMI-1640 + 10% FBS | 0.1 pM – 0.1 µM | qPCR; MTT; Cytotoxicity assays | 4–5 days | Ministry of Health (Italy) |
| 8 | Khosrokhavar et al., 2009 [50] | T47D (ER+), MDA-MB-231 (ER-) | Zearalenone & derivatives | RPMI-1640 / DMEM + 5–10% FBS | 1 pM – 1 µM | Resazurin assay | 7 days | Not specified |
| 9 | Parveen et al., 2009 [39] | MCF-7 (ER+/Luminal A) | Zearalenone & derivatives | RPMI-1640 + 10% DCC-FBS | 10 pM – 10 µM | SRB; cDNA microarray; RT-PCR; Western blot | 3 days | METI / MEXT (Japan) |
| 10 | Wang et al., 2010 [71] | MCF-7 (ER+) | Zearalenone | DMEM + 5–10% FBS | 0.01 µM – 0.02 µM | MTT; Flow cytometry; Western blot | 72 h | NSFC (China) |
| 11 | Sondergaard et al., 2012 [41] | MCF-7 (ER+) | Fusarin C | 5% FCS + Fusarin C | 0 – 100 µM | Resazurin; CALUX reporter assay | 5 days | Danish Agency for Science |
| 12 | Yun et al., 2012.[47] | MCF-7 (ER+) | Wortmannin | DMEM + 10% FBS + Insulin | 1 nM – 50 nM | CCK-8; Flow cytometry; Western blot | 24–72 h | Not specified |
| 13 | Acuña et al., 2012 [65] | MDA-MB-231 (TNBC) | (5Z)-7-Oxozeaenol | DMEM + 10% FBS | 8 µM – 10 mM | Flow cytometry; MTT; Western blot | 24 h | NCI (NIH); Pelotonia Fellowship |
| 14 | Rudolph et al., 2013 [69] | MDA-MB-231 (TNBC) | Galiellalactone, Curvularins | DMEM + 10% FCS | 0.4 µM – 80 µM | RT-qPCR; XTT; Western blot; ChIP | 48 h | Stiftung Rheinland-Pfalz |
| 15 | Jayasooriya et al., 2013 [53] | MCF-7 (ER+), MDA-MB-231 (TNBC) | Verrucarin A | RPMI-1640 + 10% FBS | 0.25 µM – 1 µM | MTT; Flow cytometry; DNA binding | 24 h | MEST (Korea) |
| 16 | Palanivel et al., 2014 [37] | MCF-7 (ER+) | Verrucarin A | RPMI-1640 + 10% FBS | 0.29 µM – 0.41 µM (IC50) | MTT; JC-1; TUNEL; Western blot | 24–48 h | University Grants Commission (India) |
| 17 | Kaya et al., 2014 [34] | MCF-7 (ER+) | α- and β-Amanitin | DMEM | 0.01 – 100 µg/mL | MTT; Biuret test | 24–36 h | Düzce University (Turkey) |
| 18 | Tseng et al., 2014 [63] | T47D (ER+), MDA-MB-453 (ER-), MDA-MB-231, MDA-MB-468 (TNBC) | Brefeldin A (BFA) | DMEM + 10% FCS | 0.05 – 1 µg/mL | WST-1; Annexin V/PI; Western blot; Clonogenic assay | 4–12 days | NSYSU-KMU; NSC (Taiwan) |
| 19 | Parandin et al., 2015 [38] | MCF-7 (ER+), MDA-MB-468 (ER-) | Zearalenone, α-Zearalenol | DMEM/F12 + 10% FBS | 1 ng/mL – 125 µg/mL | MTT colorimetric assay | 24–48 h | Ferdowsi University of Mashhad |
| 20 | Dellafiora et al., 2016 [61] | MCF-7 (ER+) | Zearalenone-14-glucoside | FBS | 1 µM | MTS assay; LC-MS | 2–6 h | Not specified |
| 21 | Li et al., 2016 [55] | BT-20, BT-474 (TNBC) | Wortmannin | DMEM/F12 + 10% FBS | Not reported | Western blot; 2-D gel electrophoresis, miRNA expression profiling | 24 h | Not specified |
| 22 | Chang et al., 2016 [49] | ZR-75-1 (ER+), MDA-MB-231 (TNBC), MCF-10A (Normal) | Cytochalasin B | DMEM / RPMI-1640 | 5 µM – 25 µM | WST-1; Flow cytometry; Fura-2; Western blot | 24 h | MOST (Taiwan) |
| 23 | Hessenberger et al., 2017 [32] | MCF-7 (ER+) | Zearalenone (ZEN) | RPMI-1640 + 10% FBS + ITS | 1 µM | Modified E-screen; WST-1 | 6 days | FFG (Austria) |
| 24 | Qi et al., 2017 [40] | MCF-7 (ER+) | Ochratoxin A (OTA) | DMEM + 10% FBS | 1 µM | SRB assay; Transepithelial transport | 1 h – 5 days | Not specified |
| 25 | Yip et al., 2017 [3] | MCF-7 (ER+) | Zearalenone, Aflatoxin B1 | DMEM/F12 + 10% FBS | 1 pM – 1 µM | MTT; Flow cytometry; qPCR | 5 days | Hong Kong University (HKU) |
| 26 | Nittoli et al., 2018 [56] | MCF-7 (ER+), MDA-MB-231 (ER-), MCF-10A (Normal) | α-Zearalenol | DMEM / RPMI-1640 + 10% FBS | 0.1 nM – 1 µM | SRB assay; Cell cycle; ROS; H-NMR | 24–72 h | Not specified |
| 27 | Tatay et al., 2018 [42] | MCF-7 (ER+) | Zearalenone, α-ZOL, β-ZOL | DMEM + 5% FBS | 6.25 µM – 25 µM | E-screen assay | 6 days | MINECO (Spain); Academy of Finland |
| 28 | Fruhauf et al., 2019 [28] | MCF-7 (ER+) | ZEN, HZEN, DHZEN | RPMI-1640 + 10% FBS | 10 pM – 10 µM | WST-1; Reporter gene assay | 18 h – 144 h | FFG (Austria) |
| 29 | Lecomte et al., 2019 [35] | MCF-7, T47D (ER+/Luminal A) | Zearalenone, Apigenin | DMEM / RPMI-1640 + 10% FBS | 0.01 µM (ZEN) | Luciferase; ChIP; TUNEL; Flow cytometry | 72 h | FUI mVolio; Inserm (France) |
| 30 | Hohenbichler et al., 2020 [33] | MCF-7 (ER+) | Alternariol (AOH), AME, ATX-I, ATX-II | DMEM + 10% FCS | 0.01 µM – 0.1 µM | EROD assay | 24 h | University of Vienna |
| 31 | Zhao et al., 2021 [59] | MDA-MB-231, MDA-MB-468 (TNBC), MCF-10A (Normal) | 10,11-Dehydrocurvularin | DMEM + 10% FBS | 2 µM – 8 µM | MTT; Flow cytometry; Annexin-V; Western blot | 30 min – 24 h | NSFC (China); Hubei Univ. |
| 32 | Kowalska et al., 2021 [60] | 184A1 (Normal Mammary) | Alternariol | DMEM + 10% FBS | 0.1 µM – 10 µM | AlamarBlue; Cell cycle; qPCR; Western blot | 24–48 h | Medical University of Lodz |
| 33 | Turkmen et al., 2021 [43] | MCF-7 (ER+) | Patulin | DMEM + 10% FCS | 1 µM – 100 µM | MTS cell viability test | 24–48 h | Inonu University (Turkey) |
| 34 | Adam et al., 2022 [59] | MCF-7 (ER+), MCF-10A (Normal) | Aflatoxin B1 (AFB1) | DMEM + FBS / Horse serum | 1.2 – 18.4 µg/mL | V-FITC Flow cytometry; DHE assay | 48 h | USM (Malaysia) |
| 35 | Zhang et al., 2023 [49] | MCF-7 (ER+) | Fusaric acid | DMEM + 10% FBS | 100 µM – 500 µM | CCK-8; Flow cytometry; ROS assay (DCFH-DA) | 24–72 h | RGC (Hong Kong); GDSTC (China) |
| 36 | Malekinejad et al., 2023 [64] | MCF-7 (ER+) | ZEN, α-ZEL, β-ZEL | DMEM | 1 nM – 0.25 µM | Pathway analysis (AhR, ERα, CYP1A1) | 48 h | Urmia Univ.; Utrecht Univ. |
| 37 | Asnaashari et al., 2023 [31] | MCF-7 (ER+) | Zearalenone | RPMI-1640 | 0.1 nM – 1 µM | Resazurin; ROS (DCFH-DA); Apoptosis | 48 h | Not specified |
| 38 | Aljazzar et al., 2023 [30] | MCF-7 (ER+) | Aflatoxin B1 (AFB1) | DMEM + 10% FBS | 10 pM – 1 µM | CCK-8; DCF-DA; qRT-PCR | 24 h | King Faisal University |
| 39 | Juan-García et al., 2024 [54] | MDA-MB-231 (ER-) | Beauvericin, Enniatin B, Ochratoxin A | DMEM + 10% FBS | 0.15 µM – 30 µM | Flow cytometry | 4–24 h | Not specified |
| 40 | Zingales et al., 2024 [58] | MDA-MB-231 (ER-) | Sterigmatocystin, Ochratoxin A, Patulin | DMEM + 10% FBS | 0.28 µM – 100 µM | 3D Cell Viability Assay; ATP assay | 24–72 h | Ministry of Science (Spain) |
| 41 | Xu et al., 2025 [52] | MCF-7, T47D (ER+); MDA-MB-231, HCC-1937 (ER-) | Zearalenone | DMEM / RPMI-1640 | 5 nM – 500 µM | qRT-PCR; RIPA | 24 h | NSFC (China); NSERC (Canada) |

The methodological parameters of the 41 included studies, detailing the specific breast cancer and non-tumorigenic cell lines utilized (e.g., ERP, ERN, TNBC, and normal mammary epithelial models), the specific mycotoxins evaluated, culture media, exposure concentration ranges, assay methodologies, duration of exposure, and funding sources, were presented.

ER+= Estrogen-receptor positive; ER-= Estrogen-receptor negative; TNBC= Triple-negative breast cancer cell line; DMEM= Dulbecco’s Modified Eagle Medium; RPMI-1640= Roswell Park Memorial Institute Medium; FBS= Fetal Bovine Serum; FCS= Fetal Calf Serum; EMEM= Eagle’s Medium Essential Medium, ROS= Reactive Oxygen Species; ZEN= Zearalenone
